# A Proposal for Future Modifications on Clinical TNM Staging System of Retinoblastoma Based on the American Joint Committee on Cancer Staging Manual, 7^th^ and 8^th^ Editions

**DOI:** 10.7150/jca.61005

**Published:** 2022-02-07

**Authors:** Yacoub A. Yousef, Ibrahim Qaddoumi, Ibrahim Al-Nawaiseh, Mona Mohammad, Dalia AlRimawi, Mario Damiano Toro, Sandrine Zweifel, Robert Rejdak, Rashed Nazzal, Mustafa Mehyar, Imad Jaradat, Iyad Sultan, Maysa Al-Hussaini

**Affiliations:** 1 Department of Surgery (Ophthalmology), King Hussein Cancer Centre, Amman, Jordan.; 2 Department of Oncology, St. Jude Children's Research Hospital, Memphis, TN, USA.; 3 Department of Biostatistics, King Hussein Cancer Centre, Amman, Jordan.; 4 Department of Ophthalmology, University of Zürich, Zürich, Switzerland.; 5 Chair and Department of General and Pediatric Ophthalmology, Medical University of Lublin, Lublin, Poland.; 6 Shami Eye Center, Amman, Jordan.; 7 Department of Radiation Oncology, King Hussein Cancer Centre, Amman, Jordan.; 8 Department of Pediatric Oncology, King Hussein Cancer Centre, Amman, Jordan.; 9 Department of Pathology and Laboratory Medicine, King Hussein Cancer Centre, Amman, Jordan.

**Keywords:** Retinoblastoma, Globe salvage, Staging system, Prognosis, American Joint Committee on Cancer

## Abstract

**Importance:** The 8^th^ edition of the American Joint Committee on Cancer (AJCC) staging manual incorporated new changes from its 7^th^ edition for classifying retinoblastoma (RB).

**Objective:** We assessed the comparative prognostic values of the 7^th^ and 8^th^ editions of the AJCC clinical (cTNM) staging manuals for RB and suggested modifications for future edition accordingly.

**Design:** A retrospective, observational study.

**Setting:** King Hussein Cancer Centre.

**Participants:** A cohort of 478 patients and 565 eyes with RB.

**Main Outcomes and Measures:** Main outcome measures included demographics; tumor features, AJCC cTNM stage, and eye salvage rates. The prognostic performance of the different staging systems was assessed with the concordance index (C-index) and likelihood ratio χ^2^ tests.

**Results:** The overall eye salvage rate was 65%. Stage migration occurred for 330 (48%) eyes with the AJCC Staging Manual, 8^th^ edition. Based on the 7^th^ edition AJCC staging, the eye salvage rate was 94% (n=177) for T1 tumors (98% for T1a, 93%for T1b, and 90%for T1c), 69% (n=204) for T2 tumors (73% for T2a and 62%for T2b), and 51% (n=40) for T3 tumors. Based on the 8^th^ edition AJCC staging, the eye salvage rate was 95% (n=139) for T1 tumors (98% for T1a and 93% for T1b), 68% (n=281) for T2 tumors (90%for T2a and 66%for T2b), and 12% (n=1) for T3 tumors. With our proposed cTNM modifications, the eye salvage rate was 94% (n=177) for T1 tumors (98%for T1a, 93%for T1b, and 90% for T1c), 66% (n=243) for T2 tumors (73% for T2a, 62% for T2b, and 55% for T2c), and 12% (n=1) for T3 tumors. As estimated by odds ratios, more advanced cTNM stage (regardless of the cTNM staging system) was significantly associated with an increased chance of treatment failure (*P* < .0001). The C-index for both the 8^th^ edition and the proposed modifications were approximately equal, and both were higher than that of the 7^th^ edition. However, the proposed modifications had the highest likelihood ratio χ^2^ value and the best bootstrap 95% confidence interval.

**Conclusions and Relevance:** Our proposed modifications on the clinical TNM Staging System for RB harbor more detailed subgroup classification criteria that provides better prognostic value for eye globe salvage than the published similar (but not identical) AJCC Staging Manual, 7^th^ and 8^th^ editions, furthermore these modifications may resolve the discrepancies in the previously published different classification systems for RB.

## Introduction

Retinoblastoma (RB) is the most common primary ocular malignant neoplasm of childhood. The prognosis for long-term survival is excellent in developed countries, where most tumors are intraocular at presentation 1,2,3. The likelihood of globe salvage depends on many tumor features, such as size, presence of subretinal fluid, vitreous seeds, and subretinal seeding 4,5.

Eye salvage rates are as high as 70% to 100% for smaller tumors (group A-C) but are as low as 23% for advanced tumors (group D or E) 6,7,8. Moreover, treatment burden increases with tumor size and severity. Although group A tumors can be treated with such focal therapies as laser or cryotherapy, large tumors or tumors with seeding require more invasive therapies, such as systemic chemotherapy, intra-arterial chemotherapy (IAC), intravitreal chemotherapy (IViC), or radioactive plaque brachytherapy 9,10,11,12,13.

The American Joint Committee on Cancer (AJCC) tumor/node/metastasis (TNM) staging system, which is a universal staging system for all cancers, included both intraocular and extraocular RB tumors in the 7^th^ edition of the AJCC Staging Manual (AJCC-7) in 2009 11. This was updated by 18 RB specialist centers from 13 countries in the 8^th^ edition of the AJCC Staging Manual (AJCC-8) in 2017 14 and is suggested to most accurately predict eye salvage, metastasis, and death. AJCC-8 incorporates intraocular (cT1-cT3) and extraocular (cT4) tumors and includes heritability, making RB the first cancer to consider heritability in its staging 15.

The AJCC-8 merged all eyes with vitreous and sub retinal seeds (regardless severity, location, and extent) in one category (cT2b), while we believe it is better for these eyes to be divided into further subgroups to give better prognostic power for this staging system. Furthermore, the AJCC staging team analyzed a heterogonous group of patients treated by different teams in different countries across the world. This heterogeneity empowered the AJCC-8, even though, we believe it should be helpful to validate this staging system based on a homogenous group of patients treated by the same team in one specialized center as well. Herein, we evaluated the discriminative ability of both the AJCC-7 and AJCC-8 clinical TNM (cTNM) staging systems for intraocular RB tumors and the effect of stage migration from AJCC-7 to AJCC-8 on the prognosis of globe salvage by performing a retrospective analysis of patients with RB who were treated at King Hussein Cancer Center (KHCC) in Amman, Jordan from 2003 to 2019. On the basis of our findings, we put forward suggestions for modifications on AJCC-8 for grouping for RB to be evaluated by different single center and multicenter studies and thereafter may be considered for future TNM staging edition to better predict the likelihood of globe salvage.

## Methods

This is a retrospective cohort study of 478 patients (697 eyes) who had clinically diagnosed RB and were treated at KHCC from 2003 to 2019. The KHCC Institutional Review Board approved this study. Selection required access to patient medical records and RetCam images. The data collected included patient demographics, treatment modalities, eye salvage, metastasis, mortality rates, and tumor features and stage at diagnosis. All tumors were restaged according to AJCC-7 and AJCC-8 criteria (eTables 1 and 2 in Supplement 1) 14,15.

### Inclusion and Exclusion Criteria

The inclusion criteria for this study comprised eyes with a clinical diagnosis of intraocular RB that received conservative treatment to avoid enucleation and/or external beam radiation therapy (EBRT). The exclusion criteria consisted of cases without follow-up records, eyes with extraocular tumor invasion, and tumors treated with primary enucleation or EBRT. We defined eye salvage as the absence of tumor activity for at least 6 months after the last active treatment.

### Tumor Features, Definitions, TNM Clinical Staging, and Treatment Modalities

We reviewed RetCam images and clinical drawings for all eyes at the time of diagnosis and documented each tumor feature. We then restaged the tumors according to AJCC-7 and AJCC-8 TNM criteria for RB (eTables 1 and 2 in Supplement 1) 14,15.

We defined intraretinal tumors as those involving the retina, without subretinal seeding or vitreous seeds. Large tumors were defined as those filling more two-thirds of the eye globe, as detected clinically, in B-scan ultrasounds, and/or in magnetic resonance images. We classified tumor seeds as subretinal or vitreous according to their location. The severity of the seeds was classified as focal vitreous and/or subretinal if fine aggregates of tumor cells were present without large clumps or “snowballs” or as massive vitreous and/or subretinal seeding if diffuse clumps or snowballs of tumor cells were present.

The standard treatment for RB consisted of a systemic chemotherapy regimen of carboplatin, vincristine, and etoposide combined with focal consolidation therapy. Each chemotherapy cycle was repeated every 3 to 4 weeks for a total of 6 to 8 cycles, according to patient condition and tumor status. Ocular oncology follow-up was provided with examination under anesthesia before each cycle of chemotherapy and every 3 to 4 weeks thereafter. Fundus photos were taken with a RetCam II instrument (Clarity Medical System, Pleasanton, CA, USA). Focal therapy was applied when needed as transpupillary thermotherapy and/or triple freeze thaw cryotherapy (MIRA CR 4000), starting after the second cycle of systemic chemotherapy. IAC, IViC, subtenon chemotherapy, and I^125^ radioactive plaque brachytherapy were used as second-line treatment options for tumor recurrence or for residual tumor activity. For this study, we defined treatment failure as the need for EBRT or enucleation.

### A Proposed Modifications on cTNM Classification System

We suggested new modifications on the cTNM classification system to be considered for future edition of the AJCC Staging Manual for RB (Table 1). Based on our suggested modifications, tumors were divided into 4 cT stages according to their clinical features. The cT1 and cT2 stages comprised potentially salvageable eyes with intraocular RB that had nearly no risk for metastasis. The cT3 stage comprised eyes with intraocular RB that were generally unsalvageable and were more likely to harbor high-risk pathologic features 15,16 and therefore had a higher risk of metastasis than cT1 and cT2 tumors. The cT4 stage comprised eyes with extraocular RB. We further divided the cT1 and cT2 stages into 3 homogenous subgroups according to their likelihood of eye salvage. The higher-stage groups and subgroups were expected to have a higher likelihood of treatment failure.

Furthermore, we divided cT3 group into 2 subgroups (Table 1). The rationale behind this modification was that cT3a in our suggested module are features that are secondary to ocular ischemia due to huge tumor size, severe RD, and**/**or secondary to increased IOP. This category of eyes are expected to have low potential for eye and vision salvage, but relatively low impact on metastatic chance as they do not invade vital structures and will not show high risk pathological features compare to the next subgroup. 16,17 In the other hand, cT3b in our suggested modifications are eyes with tumor invasion into vital structures that may cause metastasis (as ciliary body and anterior chamber), and therefore trial of eye salvage in this case will have more risk for metastasis than cT3a and may increase the mortality rate.

### Statistical Methods

The primary endpoint of this study was globe salvage. The multivariate Cox proportional hazard model was used to evaluate hazard ratios and 95% confidence intervals for the known prognostic power of all of the TNM stages. The discriminatory ability of the staging systems was measured by the concordance index (C-index). 17 The prognostic homogeneity of the staging systems was assessed with likelihood ratio χ^2^ tests. Higher C-index and likelihood ratio χ^2^ values were indicative of improved performance of the staging systems. We used SAS software v9.4 (SAS Institute Inc, Cary, NC) for statistical analyses. The odds ratio estimates for different stages were measured with a logistic regression model, and the point effect was set as group A or cT1a for the probability of treatment failure. *P* values were measured with Fisher exact tests, and values of 0.05 or less were considered significant.

## Results

### Patients Demographics and Management Outcomes

We analyzed clinical data from 478 patients: 249 (52%) were boys, and 335 (70%) had bilateral disease. We investigated 697 eyes with RB tumors: 6 had extraocular RB, 126 were treated with primary enucleation, and 565 were managed by conservative therapy targeting globe salvage. A family history of RB was present in 6 (4%) unilateral cases and in 66 (20%) bilateral cases. The median age at diagnosis was 6 and 28 months for patients with bilateral and unilateral RB, respectively.

Of 565 eyes that received conservative therapy, 421 (75%) eyes were salvaged, 369 (65%) were treated with chemoreduction alone, and 52 (9%) mandated additional therapy, such as IAC (15 eyes), IViC (21 eyes), subtenon chemotherapy (24 eyes), or I^125^ radioactive plaque therapy (13 eyes). Treatment failure occurred for 144 (25%) eyes: 130 eyes were enucleated (including 16 eyes that also received EBRT), and 14 eyes were preserved with EBRT. After a 120-month median follow-up period, 24 (5%) patients died of second neoplasms (n = 3) or metastases (n = 21).

### Tumor Features according to the AJCC-7 and AJCC-8 cTNM Staging Systems

For the 565 eyes that received conservative therapy, tumors in 488 eyes were smaller than two-thirds of the globe, and 77 were larger than two-thirds of the globe. Tumors were intraretinal in 147 eyes and extraretinal in 418 eyes. Tumor seeding was present in 368 eyes: 151 eyes had subretinal seeds, 119 eyes had vitreous seeds, and 98 eyes had both types of seeds. We detected focal seeds in 209 eyes and massive seeds in 159 eyes (Table 2).

According to AJCC-7, 189 (27%) eyes were T1 (6% were T1a, 15% were T1b, and 6% were T1c), 345 (49%) were T2 (29% were T2a and 20% were T2b), 157 (23%) were T3 (12% were T3a and 11% were T2b), and 6 were T4. According to AJCC-8, 147 (21%) eyes were T1 (6% were T1a and 15% were T1b), 471 (68%) were T2 (6% were T2a and 62% were T2b), 73 (11%) were T3, and 6 were T4 (Table 3).

### Stage Migration

Among the 691 eyes with intraocular RB, 361 (52%) had the same stage in both the AJCC-7 and AJCC-8 classification systems, including all eyes in stages cT1a and cT1b and some eyes in stages cT2a, cT2b, and cT3 (Table 4). Stage migration occurred for 330 (48%) eyes, including 246 (36%) eyes that were upstaged (ie, the stage in AJCC-8 was higher than that in the AJCC-7) and 84 (12%) that were downstaged (ie, the stage in AJCC-8 was lower than that in AJCC-7). Specifically, 42 (6%) eyes were upstaged from cT1c to cT2b, 204 (30%) upstaged from cT2a to cT2b, and 84 (12%) eyes were downstaged from cT3 to cT2b.

### Correlation between Tumor Features and Eye Salvage

We found 95% (n = 139/147) of eyes with intraretinal tumors were salvaged, whereas 67% (n = 282/418) of eyes with extraretinal tumors were salvaged. Therefore, tumor extent was a significant risk factor for treatment failure (*P* < .0001). We found 83% (n = 403/488) of eyes with small tumors were salvaged, whereas only 25% (n = 19/77) of eyes with large tumors were salvaged. Therefore, tumor size was also significant risk factor for treatment failure (*P* < .0001). The presence of tumor seeding was significant as a risk factor for failure of local control (*P <* .0001), as 90% (n = 178/197) of eyes without seeding and 66% (n = 243/368) of eyes with seeding were salvaged. In addition, tumors with massive seeds were more likely to fail treatment than those with focal seeds (*P* = .0005), as 74% (n = 154/209) eyes with focal seeds and only 46% (n = 89/159) with massive seeds were salvaged. Notably, the type of seeding did not significantly affect the salvage rate (*P* = .161), although eyes with vitreous seeds were marginally less likely to be salvaged (61%) than were those with subretinal seeds (68%) (Table 2).

### Correlation between AJCC-7 and Eye Salvage

More advanced cTNM-staged tumors had a significantly higher likelihood of treatment failure than did those with lower cTNM stages (*P* < 0.0001). The eye salvage rate was 94% (n = 177/189) for T1 tumors (98% for T1a, 93% for T1b, and 90% for T1c), 69% (n = 204/297) for T2 (73% for T2a and 62% for T2b), and 51% (n = 40/79) for T3 (55% for T3a and 12% for T2b). A logistic regression model demonstrated that the treatments for T2 and T3 tumors were 6.7 and 14 times more likely to fail than were those for T1 tumors, respectively, and the treatments for T1b, T1c, T2a, and T2b tumors were 2.8, 4.2, 14.6, and 24.5 times more likely to fail than were those for T1a tumors, respectively (Table 5).

### Correlation between AJCC-8 and Eye Salvage

More advanced cTNM-staged tumors had a significantly higher likelihood of treatment failure than did those with lower cTNM stages (*P* < .0001). The eye salvage rate was 95% (n = 139/147) for T1 tumors (98% for T1a and 93% for T1b), 68% (n = 281/410) for T2 (90% for T2a and 66% for T2b), and 12% (n = 1/8) for T3. A logistic regression model showed that the treatments for T2 and T3 tumors were 7.98 and 121.6 times more likely to fail than were those for T1 tumors, respectively, and the treatments for T1b, T2a, and T2b tumors were 2.8, 4.2, and 20.6 times more likely to fail than were those for T1a tumors, respectively (Table 5).

### A Proposal for Modifications on the cTNM Staging System

Because 95% of eyes with intraretinal tumors and 67% of those with extraretinal tumors were salvaged (*P* < .0001), we consolidated intraretinal tumors in the same stage (cT1), which we subdivided into 3 subgroups according to tumor size and severity. Because the severity of tumor seeds in extraretinal tumors affected prognosis, we assigned tumors with massive seeds as more advanced (cT2b) than tumors with focal seeds (cT2a). Only 25% of eyes with large tumors were salvaged, which was worse than eyes containing massive seeds; therefore, we assigned large tumors as cT2c. Because the type of seeding did not affect the eye salvage rate (*P* = .161), we did not consider seed type as a factor for staging. We assigned potentially unsalvageable intraocular group E tumors harboring high-risk pathologic features as stage T3 15,16 and those with extraocular invasion as stage T4.

According to our proposed cTNM staging system, 189 (27%) eyes were T1 (6% were T1a, 15% were T1b, and 6% T1c), 429 (62%) were T2 (29% were T2a, 20% were T2b, and 12% T2c), 73 (11%) were T3 (5% were T3a, and 6% were T3b), and 6 were T4 (Table 3). Advanced cTNM-staged tumors had a significantly higher likelihood of treatment failure (*P* < .0001). The eye salvage rate was 94% (n = 177/189) for T1 tumors (98% for T1a, 93% for T1b, and 90% for T1c), 66% (n = 243/368) for T2 (73% for T2a, 62% for T2b, and 55% for T2c), and 12% (n = 1/8) for T3 (17% for T3a and 0% for T2b). Logistic regression demonstrated that the treatments for T2 and T3 tumors were 6.1 and 103 times more likely to fail than were those for T1 tumors, respectively, and the treatments for T1b, T1c, T2a, T2b, and T2c tumors were 2.8, 4.2, 14.6, 24.5, and 32.8 times more likely to fail than were those for T1a tumors, respectively (Table 5).

**Figure 1:**
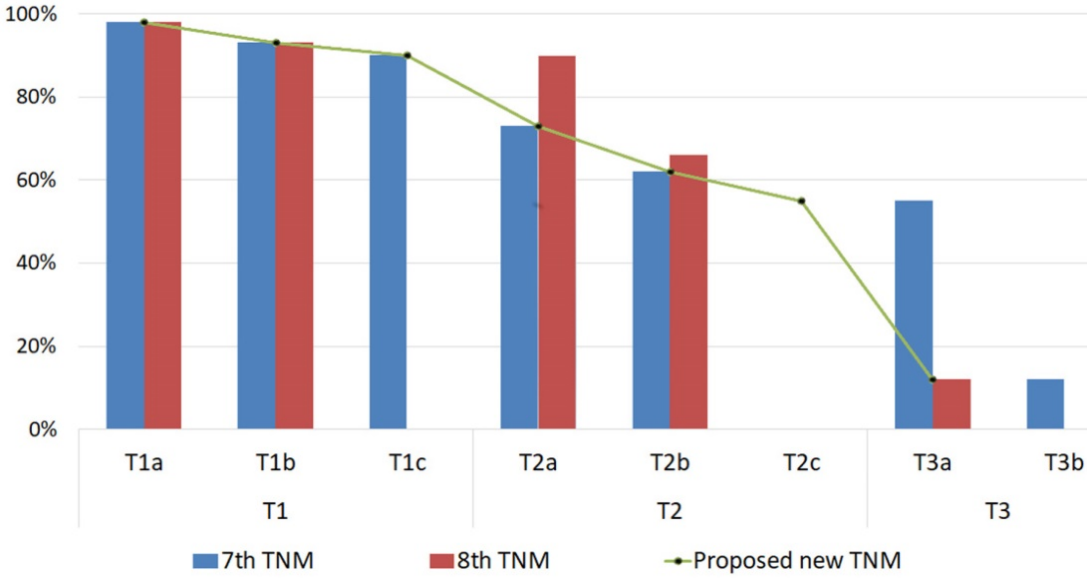
Globe Salvage Rates of Eyes with Intraocular Retinoblastoma. The globe salvage rates are shown for tumors classified with the American Joint Committee on Cancer Staging Manual, 7^th^ edition and 8^th^ editions and our proposed cTNM staging system modifications.

The 3 TNM staging systems (AJCC-7, AJCC-8, and the modified staging system) we investigated were all generally able to predict the likelihood of eye salvage (Figure 1 and Table 5). We compared the performance of AJCC-7, AJCC-8, and the modified staging system by calculating the C-index and likelihood ratio χ^2^ values (Table 5). The modified staging system had the highest likelihood ratio χ^2^ value. The C-index values for both AJCC-8 and the proposed system were approximately equal (both were higher than that of AJCC-7), but the confidence interval for the proposed staging system was (0.67-0.77) which is better than the confidence interval for AJCC-8 (0.62-0.87), indicating that our proposed staging system has better prognostic capability than AJCC-8.

### Correlation between AJCC-7, AJCC-8, and the proposed modifications and the Rate of Metastasis

**Figure 2:**
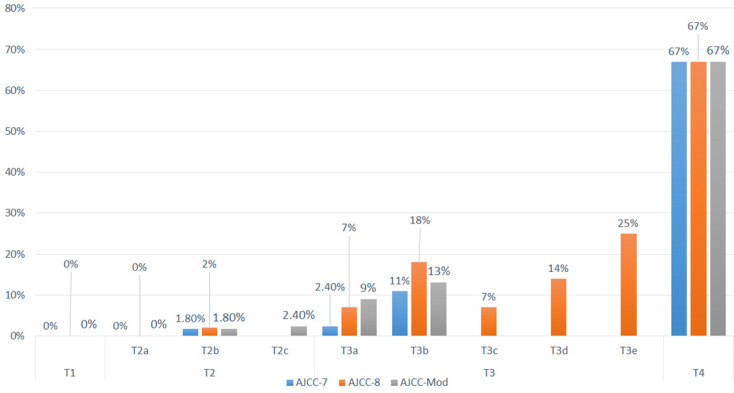
Metastatic Rates of Patients with Retinoblastoma. The metastatic rates are shown for tumors classified with the American Joint Committee on Cancer Staging Manual, 7^th^ edition and 8^th^ editions and our proposed cTNM staging system modifications.

Twenty-one patients developed metastasis; no patient with the worst eye stage T1 developed metastasis in any of the three TNM staging systems. Four patients had extraocular RB and were stage T4 in the three systems, and eight patients had the worst eye staged as T3b based on AJCC-7, and were staged T3 based on both AJCC-8 and the proposed modifications (Table 6). Because of the low number of patients with metastasis in each subgroup, no statistical correlation could be done (Figure 2).

## Discussion

Accurate staging is essential to guide treatment and predict prognosis 18,19. When we evaluated the discriminative ability of AJCC-7 and AJCC-8 to predict the likelihood of globe salvage, we found that higher cT stage in both editions was associated with an increased risk of treatment failure. However, our proposed staging system exhibited the best performance of the 3, indicating that our proposed modifications are better for predicting globe salvage.

Recently, the predictive value of the 8^th^ edition AJCC staging for RB for both survival and eye globe salvage was evaluated by 8 ophthalmic oncology centers from 13 countries over 6 continents. They found that the overall eye globe salvage rate without EBRT was 52%, and the cumulative 5- year survival and eye globe salvage estimates by clinical TNM categories were 100% and 96% for category cT1a, 98% and 88% for cT1b, 98% and 60% for cT2a, 96% and 57% for cT2b, 89% and 25% for cT3 tumors, respectively 20,21. Based on that large and heterogenous RB patient population, the 8^th^ edition AJCC RB Staging System was found to be capable of predicting metastasis-related mortality as well as eye globe-salvage 20,21. In our study we analyzed a homogenous group of RB patients who were treated by the same team in a single center, and similarly, we found that the eye salvage rate was 95% for T1 tumors (98% for T1a and 93% for T1b), 68% for T2 tumors (90% for T2a and 66% for T2b), and 12% for T3 tumors. Base on that, we looked for more accurate and detailed clinical features that may harbor higher predictive power for eye globe salvage and we incorporated that in our proposed modified staging system.

We divided cT2 tumors into T2a to T2b subgroups according to the presence or absence of tumor seeds, which was supported by our finding that 90% of eyes without tumor seeding were salvaged. However, the cT2b subgroup encompassed a large group of heterogeneous tumors because this subgroup included all tumors with seeding, regardless of their location (subretinal vs vitreal) or severity (focal seeds vs massive seeds). The American Joint Committee on Cancer Ophthalmic Oncology Task analyzed 592 eyes in group cT2b (AJCC-8) that had complete data for globe salvage analysis 5. They found that the 5-year Kaplan-Meier cumulative globe-salvage was 78% for eyes with focal seeding and 49% for eyes with diffuse seeding, and Cox proportional hazards regression analysis confirmed a higher local treatment failure risk with diffuse seeds as compared with focal seeds (hazard rate: 2.8; p<0.001) 5. In the other hand there was insufficient evidence to prove or disprove an association between tumor seeds type and local treatment failure risk (p=0.06). Similarly, when we analyzed 368 T2b (AJCC-8) eyes that received conservative therapy, we found that tumors with massive seeds were more likely to fail treatment than those with focal seeds, as 74% of eyes with focal seeds in our series were salvaged, while only 46% of eyes with massive seeds were salvaged (*P* = 0.0005). Notably, in our series the type of seeding did not significantly affect the salvage rate (*P* = 0.161), although eyes with vitreous seeds were marginally less likely to be salvaged (61%) than were those with subretinal seeds (68%).

Although international RB staging systems can successfully predict the likelihood of eye salvage with intravenous chemotherapy 6,7,8, the effect of tumor size on globe salvage prognosis remains controversial in the ocular oncology community. Two published versions of an international RB classification system are widely used: the International Intraocular Retinoblastoma Classification (IIRC) 4 and the Intraocular Classification for Retinoblastoma (ICRB) 5. Although both systems are very similar, they contain subtle differences, most notably in their classification of group E eyes. The ICRB considers large tumors (ie, > 50% of the globe) as group E, whereas these tumors would be assigned as group B, C, or D by the IIRC. Because of these discrepancies, the international staging system is inconsistent, which is also the case for AJCC-7 and AJCC-8. AJCC-7 upstages tumors from T2 to T3a if the tumors fill more than two-thirds of the globe and to T3b if the tumors are associated with destroyed eye structures, such as neovascular or angle closure glaucoma, tumor extension into the anterior segment, hyphema, vitreous hemorrhage, or orbital cellulites. Because AJCC-8 excludes tumor size from cT3 grouping, group cT3 tumors have only the features of group E tumors, according to the IIRC 4. To resolve this issue, we propose modifications on T2 grouping in AJCC-8, and we considered specific subgroup for tumors occupying more than two-thirds of the globe (cT2c). The likelihood of eye salvage for these large tumors was 25%, which is better than the unsalvageable cT3 (IIRC group E) tumors but still worse than small tumors with focal or massive seeds (T2a and T2b).

We believe it is important to consider IIRC group E tumors (stage cT3 in AJCC-8) as a solitary group because although they comprise intraocular tumors, these tumors are at higher risk of treatment failure than are T1 and T2 tumors. These tumors harbor high-risk histopathologic features that predispose them to an increased risk of systemic metastasis 15,22,23,24,25.

In our proposal, we classified cT3 group into 2 subgroups; cT3a are eyes with features secondary to ocular ischemia (including neovascular glaucoma, buphthalmous, hyphaema, and vitreous hemorrhage), and cT3b are eyes with tumor invasion into vital structures that may cause metastasis (including ciliary body and anterior chamber invasion, phthisis, and orbital cellulitis).

cT3a eyes are expected to have low potential for eye and vision salvage because of ischemia, but relatively lower chance of metastasis as they do not invade vital structures and will not show high risk pathological features in comparison to cT3b eyes 16, 17. The low number of patients in group cT3 in our study did not help us to prove our theory about dividing cT3 eyes into 2 subgroups. In the literature, there is a significant disparity regarding the risk of metastasis associated with high-risk clinical features. Chantada et al and Kim et al suggested that glaucoma is significantly associated with high-risk pathology. In these reports, authors compared the chance of HRPF between eyes with glaucoma and eyes without glaucoma, but they did not compare that with eyes with anterior segment invasion and/or phthisis 26, 27. Similarly, Kashyap et al concluded that vitreous hemorrhage, hyphema, staphyloma, and orbital cellulitis were predictors of high-risk pathology, but again in comparison with less advanced tumors 28. Furthermore, eyes with hyphaema will be more difficult to assess for anterior chamber and ciliary body invasion clinically unless they used specific imaging studies as ultrasound biomicroscopy. Based on that, there is no evidence until now about which clinical signs of advanced intraocular RB are associated with worse prognosis than others. Hopefully bigger international studies may be done in the future to validate dividing cT3 into 2 subgroups or more that show difference in survival, and that be with or against our theory.

## Strengths and Limitations

The same clinical team consistently managed all of the tumors in our analysis, and the sample size and tumor diversity in our cohort yielded statistical significance, strengthening our study. The retrospective nature, analysis of only intraocular RB tumors, and omission of survival prognosis were limitations in our study. Furthermore, our results are single-center data that may be not directly comparable to that derived from patients around the world.

## Conclusions

The clinical staging criteria used in the AJCC-7 and AJCC-8 TNM staging systems can predict the likelihood of globe salvage with conservative treatment. Tumors with massive seeding and large tumors are best considered as a more advanced stage. Accordingly, we propose modifications that outperform the current cTNM staging system and may resolve the discrepancies in previously published RB classification systems.

## Supplementary Material

Supplementary tables.

## Figures and Tables

**Table 1:** Proposal for Modifications on the Definition of cT in the AJCC cTNM Staging Classification System for Retinoblastoma^a^

Stage	Tumor Characteristics
**cTX**	Unknown evidence of intraocular tumor
**cT0**	No evidence of intraocular tumor
**cT1**	Intraretinal tumor(s) occupying < 2/3 globe, with subretinal fluid ≤ quadrant of the globe; No vitreous or subretinal seeding is allowed
cT1a	Tumors ≤ 3 mm and further than 1.5 mm from the disc and fovea
cT1b	Tumors > 3 mm or closer than 1.5 mm to the disc and fovea; No retinal detachment or subretinal fluid beyond 5 mm from the base of the tumor
cT1c	Subretinal fluid > 5 mm from the base of any tumor and up to quadrant of the globe
**cT2**	Intraocular tumor(s) with retinal detachment, vitreous seeding, or subretinal seeding
cT2a	Tumors with focal vitreous seeding and/or subretinal seeding Retinal detachment > quadrant of the globe but < total
cT2b	Tumors with massive vitreous seeding and/or subretinal seeding and/or total exudative retinal detachment
cT2c	Large intraocular tumors occupying more than 2/3 of the eye globe
**cT3**	Advanced intraocular tumor(s)
cT3a	Raised intraocular pressure with neovascularization and/or buphthalmos; Hyphema and/or massive vitreous hemorrhage
cT3b	Tumor invasion of the pars plana, ciliary body, lens, zonules, iris or anterior chamber; Phthisis or pre-phthisis bulbi or aseptic orbital cellulitis
**cT4**	Extraocular tumor(s) involving the orbit, including the optic nerve
cT4a	Radiological evidence of retrobulbar optic nerve involvement or thickening of the optic nerve or involvement of the orbital tissues
cT4b	Extraocular tumor clinically evident with proptosis and orbital mass

Abbreviations: AJCC, American Joint Committee on Cancer; cTNM, clinical tumor/node/metastasis. ^a^Intraretinal tumors were defined as tumors involving the retina without subretinal seeding or vitreous seeds. Focal vitreous and/or subretinal seeding were defined as fine aggregates of tumor cells, and massive vitreous and/or subretinal seeding were defined as diffuse clumps or “snowballs” of tumor cells are present. Large tumors were defined as tumors filling more 2/3 of the eye globe, as detected clinically, in B-scan ultrasounds, and/or with magnetic resonance imaging.

**Table 2:** Association of Tumor Features and Globe Salvage

Tumor Features	Conservative Therapy	Eye Salvage^b^ (%)		*P* value
Total no. eyes^a^	565	421	75	
**Tumor extent**				< .0001
Intraretinal	147	139	95	
Extraretinal	418	282	67	
**Tumor size**				< .0001
Small (< 2/3 of the globe)	488	403	83	
Large (> 2/3 of the globe)	77	19	25	
**Presence of seeds**				< .0001
No	197	178	90	
Yes	368	243	66	
**Type of seeds**				.161
Subretinal	151	103	68	
Vitreous	119	73	61	
Combined	98	67	68	
**Severity of seeds**				.0005
Focal	209	154	74	
Massive	159	89	46	

^a^We evaluated data from 478 patients, with 813 total affected eyes. Because 116 eyes were enucleated before referral to our center the number of affected eyes treated was 697, and 565 tumors were treated by conservative therapy. ^b^This is the number of eyes that were salvaged without external beam radiation therapy or enucleation.

**Table 3:** Tumor cTNM Staging and Management Outcomes

	Number (%)	Primary Enucleation (%)	Amended Treatment	Overall Eye Salvage for Amended Treatment
Total no. eyes^a^	697	132 (19)	565	421 (75)
***AJCC-7***				
**T1**	189 (27)	0 (0)	189	177 (94)
T1a	41 (6)	0 (0)	41	40 (98)
T1b	106 (15)	0 (0)	106	99 (93)
T1c	42 (6)	0 (0)	42	38 (90)
**T2**	345 (49)	48 (14)	297	204 (69)
T2a	204 (29)	26 (13)	176	129 (73)
T2b	141 (20)	22 (16)	121	75 (62)
**T3**	157 (23)	78 (50)	79	40 (51)
T3a	84 (12)	13 (16)	71	39 (55)
T3b	73 (11)	65 (89)	8	1 (12)
T4^b^	6 (<1)	6 (100)	0	0 (0)
***AJCC-8***				
**T1**	147 (21)	0 (0)	147	139 (95)
T1a	41 (6)	0 (0)	41	40 (98)
T1b	106 (15)	0 (0)	106	99 (93)
**T2**	471 (68)	61 (13)	410	281 (68)
T2a	42 (6)	0 (0)	42	38 (90)
T2b	429 (62)	61 (14)	368	243 (66)
**T3**	73 (11)	65 (89)	8	1 (12)
T3a	23 (3)	21 (91)	2	0 (0)
T3b	11 (2)	10 (91)	1	0 (0)
T3c	28 (4)	24 (86)	4	1 (25)
T3d	7 (1)	6 (86)	1	0 (0)
T3e	4 (1)	4 (100)	0	0 (0)
	6 (<1)	6 (100)	0	0 (0)
**T4**	6 (<1)	6 (100)	0	0 (0)
T4a	5 (<1)	5 (100)	0	
T4b	1 (<1)	1 (100)	0	
***Proposed Modified cTNM Staging System***				
**T1**	189 (27)	0 (0)	189	177 (94)
T1a	41 (6)	0 (0)	41	40 (98)
T1b	106 (15)	0 (0)	106	99 (93)
T1c	42 (6)	0 (0)	42	38 (90)
**T2**	429 (62)	61 (14)	368	243 (66)
T2a	204 (29)	26 (13)	176	129 (73)
T2b	141 (20)	22 (16)	121	75 (62)
T2c	84 (12)	13 (16)	71	39 (55)
**T3**	73 (11)	65 (89)	8	1 (12)
T2a	35 (5)	30 (86)	5	1 (20)
T2b	38 (6)	35 (92)	3	0 (0)
**T4**	6 (<1)	6 (100)	0	0 (0)
Metastasis^c^	22 (4)			
Secondary malignancy	4 (1)			
Mortality^d^	24 (5)			

Abbreviations: AJCC-7, American Joint Committee on Cancer Staging Manual, 7th edition; AJCC-8, American Joint Committee on Cancer Staging Manual, 8th edition; cTNM, clinical tumor/node/metastasis. ^a^ We evaluated data from 478 patients, with 813 total affected eyes. Because 116 eyes were enucleated before referral to our center, the number of affected eyes treated was 697. ^b^ Of 126 eyes treated with primary enucleation, 6 had extraocular disease treated by enucleation after neoadjuvant chemotherapy. ^c^ This included all patients with metastasis including those who had metastasis at time of diagnosis and those who developed metastasis during therapy and follow up at our center. All but one died because of metastasis. ^d^ Twenty four patients died at the last date of follow up; 21 with metastasis and 3 with second malignancy.

**Table 4:** Distribution of Tumors according to the AJCC cTNM Staging Manual, 7^th^ and 8^th^ Editions

	AJCC-8 cTNM Stage					Sum
	1a	1b	2a	2b	3	
**AJCC-7 cTNM Stage**						
1a	41	0	0	0	0	41
1b	0	106	0	0	0	106
1c	0	0	42	0	0	42
2a	0	0	0	204	0	204
2b	0	0	0	141	0	141
3	0	0	0	84	73	157
**Sum**	41	106	42	429	73	691

Abbreviations: Abbreviations: AJCC-7, American Joint Committee on Cancer Staging Manual, 7th edition; AJCC-8, American Joint Committee on Cancer Staging Manual, 8^th^ edition; cTNM, clinical tumor/node/metastasis.

**Table 5:** Prognostic Performance of AJCC-7, AJCC-8, and the Proposed Modifications on cTNM Staging Systems

	Concordance Indices				Likelihood Ratio χ^2^
	C-index		Bootstrap 95% CI		
AJCC-7	0.6927		0.6391-0.7464		(29.51) < .0001
AJCC-8	0.7454		0.6203-0.8706		(18.49) < .0001
Proposed System	0.7249		0.6714-0.7785		(42.71) < .0001
			**Odds Ratio Estimates**		
	**Eye Salvage (%)**	**Effect**	***P* Value**	**Point Estimate**	**95% Wald Confidence Limits**
***AJCC-7***					
**T1**	177 (94)	-			
T1a	40 (98)	-			
T1b	99 (93)	T1b vs T1a	.3381	2.828	0.337-23.732
T1c	38 (90)	T1c vs T1a	.2076	4.210	0.450-39.384
**T2**	204 (69)	T2 vs T1	< .0001	6.724	3.567-12.676
T2a	129 (73)	T2a vs T1a	.0091	14.573	1.948-109.002
T2b	75 (62)	T2b vs T1a	.0019	24.533	3.261-184.554
**T3**	40 (51)	T3 vs T1	< .0001	14.381	6.914-29.912
T3a	39 (55)	T3a vs T1a	.0008	32.819	4.273-252.050
T3b	1 (12)	T3bvs T1a	.0001	279.991	15.627 - >999.999
***AJCC-8***					
**T1**	139 (95)	-			
T1a	40 (98)	-			
T1b	99 (93)	T1b vs T1a	.3381	2.828	0.337-23.732
**T2**	281 (68)	T2 vs T1	< .0001	7.976	3.796-16.758
T2a	38 (90)	T2a vs T1a	.2076	4.21	0.45-39.384
T2b	243 (66)	T2b vs T1a	.003	20.575	2.796-151.422
**T3**	1 (12)	T3 vs T1	< .0001	121.615	13.299 - >999.999
***Proposed Modified cTNM Staging System***					
**T1**	177 (94)	-			
T1a	40 (98)	-			
T1b	99 (93)	T1b vs T1a	.3381	2.828	0.337-23.732
T1c	38 (90)	T1c vs T1a	.2076	4.210	0.450-39.384
**T2**	243 (66)	T2 vs T1	< .0001	6.127	3.275-11.461
T2a	129 (73)	T2a vs T1a	.0091	14.573	1.948-109.002
T2b	75 (62)	T2b vs T1a	.0019	24.533	3.261-184.554
T2c	39 (55)	T2c vs T1a	.0008	32.819	4.273-252.050
**T3**	1 (12)	T3 vs T1	< .0001	103.247	11.726-909.088

Abbreviations: AJCC-7, American Joint Committee on Cancer Staging Manual, 7th edition; AJCC-8, American Joint Committee on Cancer Staging Manual, 8th edition; cTNM, clinical tumor/node/metastasis.

**Table 6:** Tumor cTNM Staging and Metastasis related Mortality (478 RB patients)^a^

	Number (%)	Metastasis	Metastasis Related Mortality
Total no. patients^b^ with known stage	362 patients	17 (5)	16 (4.4)
Unknown stage	116 patient	4	4
***AJCC-7***			
**T1**	**20 (5.5)**	**0 (0)**	**0 (0)**
T1a	5 (1.4)	0 (0)	0 (0)
T1b	10 (2.4)	0 (0)	0 (0)
T1c	5 (1.4)	0 (0)	0 (0)
**T2**	**179 (50)**	**3 (1.7)**	**3 (1.7)**
T2a	19 (5)	0 (0)	0 (0)
T2b	160 (44)	3(1.8)	3(1.8)
**T3**	**157 (43)**	**10 (6.4)**	**9 (5.7)**
T3a	84 (23)	2 (2.4)	2 (2.4)
T3b	73 (20)	8 (11)	7 (9.5)
**T4^b^**	**6 (1.6)**	**4 (67%)**	**4 (67%)**
***AJCC-8***			
**T1**	**15 (4)**	**0 (0)**	**0 (0)**
T1a	5 (1.4)	0 (0)	0 (0)
T1b	10 (2.8)	0 (0)	0 (0)
**T2**	**268 (74)**	**5 (2)**	**5 (2)**
T2a	5 (1.4)	0 (0)	0 (0)
T2b	263 (73)	5 (2)	5 (2)
**T3**	**73 (20)**	**8 (11)**	**7 (9.5)**
T3a	23 (3)	2 (7)	2 (7)
T3b	11 (2)	2 (18)	2 (18)
T3c	28 (4)	2 (7)	1(4)
T3d	7 (1)	1(14)	1(14)
T3e	4 (1)	1(25)	1(25)
**T4**	**6 (1.6)**	**4 (67%)**	**4 (67%)**
T4a	5 (<1)	3	3
T4b	1 (<1)	1	1
**Proposed Modified cTNM Staging System**			
**T1**	**20 (5.5)**	0 (0)	0 (0)
T1a	5 (1.4)	0 (0)	0 (0)
T1b	10 (2.8)	0 (0)	0 (0)
T1c	5 (1.4)	0 (0)	0 (0)
**T2**	**263 (73)**	5 (2)	5 (2)
T2a	19 (5.3)	0 (0)	0 (0)
T2b	160 (44)	3(1.8)	3(1.8)
T2c	84 (23)	2 (2.4)	2 (2.4)
**T3**	**73 (20)**	**8 (11)**	**7 (9.5)**
T2a	35 (5)	3 (9)	2 (6)
T2b	38 (6)	5 (13)	5 (13)
**T4**	**6 (1.4)**	4 (67%)	4 (67%)
Metastasis^b^	**22/478 (4.6)**		
Metastasis related Mortality	21 (4.4%)		

Abbreviations: AJCC-7, American Joint Committee on Cancer Staging Manual, 7th edition; AJCC-8, American Joint Committee on Cancer Staging Manual, 8th edition; cTNM, clinical tumor/node/metastasis. ^a^ We correlated between the metastasis and the stage of the worst affected eye for patients with bilateral disease. ^b^ We evaluated data from 478 patients, 116 patients had one eye enucleated before referral to our center, therefore the stage of the worst eye wasn't known. ^b^ This included all patients with metastasis including those who had metastasis at time of diagnosis and those who developed metastasis during therapy and follow up at our center.
