# Supplementary material for: A Proposal for Future Modifications on Clinical TNM Staging System of Retinoblastoma Based on the American Joint Committee on Cancer Staging Manual, 7th and 8th Editions
Source: J Cancer. 2022 Feb 7;13(4):1336–45. doi: 10.7150/jca.61005 (PMC8899378; doi:10.7150/jca.61005)
Supplement: Supplementary file 1 — Supplementary tables. [file jcav13p1336s1.pdf]

Supplementary Table 1: Definition of primary tumour (cT) in The 7th edition, American Joint Committee on Cancer (AJCC) staging for Retinoblastoma.[13]		
cTX		Primary tumor cannot be assessed
cT0		No evidence of primary tumor
cT1		Tumors no more than 2/3 the volume of the eye with no vitreous or subretinal seeding
	cT1a	No tumor in either eye is greater than 3 mm in largest dimension or located closer than 1.5 mm to the optic nerve or fovea
	cT1b	At least one tumor is greater than 3 mm in largest dimension or located closer than 1.5 mm to the optic nerve or fovea. No retinal detachment or subretinal fluid beyond 5 mm from the base of the tumor
	cT1c	At least one tumor is greater than 3 mm in largest dimension or located closer than 1.5 mm to the optic nerve or fovea, with retinal detachment or subretinal fluid beyond 5 mm from the base of the tumor
cT2		Tumors no more than 2/3 the volume of the eye with vitreous or subretinal seeding. Can have retinal detachment
	cT2a	Focal vitreous and/or subretinal seeding of fi ne aggregates of tumor cells is present, but no large clumps or “snowballs” of tumor cells
	cT2b	Massive vitreous and/or subretinal seeding is present, defined as diffuse clumps or “snowballs” of tumor cells
cT3		Severe intraocular disease
	cT3a	Tumor fills more than 2/3 of the eye
	cT3b	One or more complications present, which may include tumor-associated neovascular or angle closure glaucoma, tumor extension into the anterior segment, hyphema, vitreous hemorrhage, or orbital cellulitis
cT4		Extraocular disease detected by imaging studies
	cT4a	Invasion of optic nerve

	cT4b	Invasion into the orbit
	cT4c	Intracranial extension not past chiasm
	cT4d	Intracranial extension past chiasm
**Definition of regional lymph nodes (cN)**		
cNX		Regional lymph nodes cannot be assessed
cN0		No regional lymph nodes involvement
cN1		Regional lymph node involvement (preauricular, cervical, submandibular)
cN2		Distant lymph node involvement
**Definition of distant metastasis (M)**		
cM0		No metastasis
cM1		Systemic metastasis
	cM1a	Single lesion to sites other than CNS
	cM1b	Multiple lesions to sites other than CNS
	cM1c	Prechiasmatic CNS lesion(s)
	cM1d	Postchiasmatic CNS lesion(s)
	cM1e	Leptomeningeal and/or CSF involvement
** From Chapter 44: Retinoblastoma. In: Edge SD, Byrd DR, Carducci MA, Compton CC, eds. AJCC Cancer Staging Manual. 7th ed. New York, NY: Springer; 2009.*		

**Supplementary Table 2: Definition of primary tumour (cT) in The 8th edition, American Joint Committee on Cancer (AJCC) staging for Retinoblastoma** [14]		
cTX		Unknown evidence of intraocular tumour
cT0		No evidence of intraocular tumour
cT1		Intraocular tumour(s) with sub-retinal fluid ≤ 5mm from the base of any tumour
	cT1a	Tumours ≤ 3mm and further than 1.5 mm from the disc and fovea
	cT1b	Tumours > 3 mm or closer than 1.5 mm to the disc and fovea
cT2		Intraocular tumour(s) with retinal detachment, vitreous seeding or sub-retinal seeding
	cT2a	Sub-retinal fluid > 5 mm from the base of any tumour
	cT2b	Tumours with vitreous seeding and/or sub-retinal seeding
cT3		Advanced intraocular tumour(s)
	cT3a	Phthisis or pre-phthisis bulbi
	cT3b	Tumour invasion of the pars plana, ciliary body, lens, zonules, iris or anterior chamber
	cT3c	Raised intraocular pressure with neovascularization and/or buphthalmos
	cT3d	Hyphema and/or massive vitreous hemorrhage
	cT3e	Aseptic orbital cellulitis
cT4		Extraocular tumour(s) involving the orbit, including the optic nerve
	cT4a	Radiological evidence of retrobulbar optic nerve involvement or thickening of the optic nerve or involvement of the orbital tissues
	cT4b	Extraocular tumour clinically evident with proptosis and orbital mass

**Definition of regional lymph nodes (cN)**		
cNX		Regional lymph nodes cannot be assessed
cN0		No regional lymph nodes involvement
cN1		Evidence of preauricular, submandibular, and cervical lymph node involvement
**Definition of distant metastasis (M)**		
cM0		No signs or symptoms of intracranial or distant metastasis
cM1		Distant metastasis without microscopic confirmation
	cM1a	Tumour(s) involving any distant site (e.g. bone marrow, liver) on clinical or radiological tests
	cM1b	Tumour involving the central nervous system on radiological imaging (not including trilateral retinoblastoma)
pM1		Distant metastasis with microscopic confirmation
	pM1a	Histopathological confirmation of tumour at any distant site (e.g. bone marrow, liver, or other)
	pM1b	Histopathological confirmation of tumour in the cerebrospinal fluid or CNS parenchyma
**Definition of heritable trait (H)**		
HX		Unknown or insufficient evidence of a constitutional RB1 gene mutation
H0		Normal RB1 alleles in blood tested with demonstrated high sensitivity assays
H1		Bilateral retinoblastoma, retinoblastoma with an intracranial CNS midline embryonic tumour (i.e. trilateral retinoblastoma), patient with family history of retinoblastoma, or molecular definition of constitutional RB1 gene mutation
* *From Mallipatna AC, Gallie BL, Chévez-Barrios P, et al. Retinoblastoma. In: Amin MB, Edge SB, Greene FL, Byrd DR, Brookland RK, Washington MK, et al., editors. AJCC Cancer Staging Manual. 8th ed. New York: Springer; 2017. pp. 819-831.*		
